# Laparoscopic management of diaphragmatic eventration: a three-step procedure of diaphragm reconstruction

**DOI:** 10.1093/gastro/goae043

**Published:** 2024-05-06

**Authors:** Bing Zeng, Wenchang Gan, Taicheng Zhou, Shuang Chen, Enmin Huang, Zhilong Yuan, Fuheng Liu, Zhiqiang Liang, Yingru Li

**Affiliations:** Department of General Surgery, Hernia and Abdominal Wall Surgery, The Sixth Affiliated Hospital, Sun Yat-sen University, Guangzhou, Guangdong, P. R. China; Guangdong Provincial Key Laboratory of Colorectal and Pelvic Floor Diseases, The Sixth Affiliated Hospital, Sun Yat-sen University, Guangzhou, Guangdong, P. R. China; Biomedical Innovation Center, The Sixth Affiliated Hospital, Sun Yat-sen University, Guangzhou, Guangdong, P. R. China; Department of General Surgery, Hernia and Abdominal Wall Surgery, The Sixth Affiliated Hospital, Sun Yat-sen University, Guangzhou, Guangdong, P. R. China; Guangdong Provincial Key Laboratory of Colorectal and Pelvic Floor Diseases, The Sixth Affiliated Hospital, Sun Yat-sen University, Guangzhou, Guangdong, P. R. China; Biomedical Innovation Center, The Sixth Affiliated Hospital, Sun Yat-sen University, Guangzhou, Guangdong, P. R. China; Department of General Surgery, Hernia and Abdominal Wall Surgery, The Sixth Affiliated Hospital, Sun Yat-sen University, Guangzhou, Guangdong, P. R. China; Guangdong Provincial Key Laboratory of Colorectal and Pelvic Floor Diseases, The Sixth Affiliated Hospital, Sun Yat-sen University, Guangzhou, Guangdong, P. R. China; Biomedical Innovation Center, The Sixth Affiliated Hospital, Sun Yat-sen University, Guangzhou, Guangdong, P. R. China; Department of General Surgery, Hernia and Abdominal Wall Surgery, The Sixth Affiliated Hospital, Sun Yat-sen University, Guangzhou, Guangdong, P. R. China; Guangdong Provincial Key Laboratory of Colorectal and Pelvic Floor Diseases, The Sixth Affiliated Hospital, Sun Yat-sen University, Guangzhou, Guangdong, P. R. China; Biomedical Innovation Center, The Sixth Affiliated Hospital, Sun Yat-sen University, Guangzhou, Guangdong, P. R. China; Department of General Surgery, Hernia and Abdominal Wall Surgery, The Sixth Affiliated Hospital, Sun Yat-sen University, Guangzhou, Guangdong, P. R. China; Guangdong Provincial Key Laboratory of Colorectal and Pelvic Floor Diseases, The Sixth Affiliated Hospital, Sun Yat-sen University, Guangzhou, Guangdong, P. R. China; Biomedical Innovation Center, The Sixth Affiliated Hospital, Sun Yat-sen University, Guangzhou, Guangdong, P. R. China; Department of General Surgery, Hernia and Abdominal Wall Surgery, The Sixth Affiliated Hospital, Sun Yat-sen University, Guangzhou, Guangdong, P. R. China; Guangdong Provincial Key Laboratory of Colorectal and Pelvic Floor Diseases, The Sixth Affiliated Hospital, Sun Yat-sen University, Guangzhou, Guangdong, P. R. China; Biomedical Innovation Center, The Sixth Affiliated Hospital, Sun Yat-sen University, Guangzhou, Guangdong, P. R. China; Department of General Surgery, Hernia and Abdominal Wall Surgery, The Sixth Affiliated Hospital, Sun Yat-sen University, Guangzhou, Guangdong, P. R. China; Guangdong Provincial Key Laboratory of Colorectal and Pelvic Floor Diseases, The Sixth Affiliated Hospital, Sun Yat-sen University, Guangzhou, Guangdong, P. R. China; Biomedical Innovation Center, The Sixth Affiliated Hospital, Sun Yat-sen University, Guangzhou, Guangdong, P. R. China; Department of General Surgery, Hernia and Abdominal Wall Surgery, The Sixth Affiliated Hospital, Sun Yat-sen University, Guangzhou, Guangdong, P. R. China; Guangdong Provincial Key Laboratory of Colorectal and Pelvic Floor Diseases, The Sixth Affiliated Hospital, Sun Yat-sen University, Guangzhou, Guangdong, P. R. China; Biomedical Innovation Center, The Sixth Affiliated Hospital, Sun Yat-sen University, Guangzhou, Guangdong, P. R. China; Department of General Surgery, Hernia and Abdominal Wall Surgery, The Sixth Affiliated Hospital, Sun Yat-sen University, Guangzhou, Guangdong, P. R. China; Guangdong Provincial Key Laboratory of Colorectal and Pelvic Floor Diseases, The Sixth Affiliated Hospital, Sun Yat-sen University, Guangzhou, Guangdong, P. R. China; Biomedical Innovation Center, The Sixth Affiliated Hospital, Sun Yat-sen University, Guangzhou, Guangdong, P. R. China

## Introduction

Diaphragmatic eventration (DE) is a rare condition with an incidence rate of approximately 0.05% [1]. Anatomically, its characteristic feature is the uninterrupted integrity of the diaphragmatic muscle, maintaining normal connections with the sternum, ribs, and vertebral column of the lower back [2]. Currently, there is no consensus on the optimal surgical treatment for DE. Traditional surgical approaches include transthoracic or transabdominal diaphragmatic plication [3, 4]. Recent studies have also reported the utilization of laparoscopic diaphragmatic resection with endostaplers in the treatment of DE [5]. Here we report a case of left posterior DE successfully treated using a three-step procedure of laparoscopic diaphragm reconstruction.

## Case report

The procedure was performed on a 41-year-old male patient who had been diagnosed with DE. The patient was referred to our medical center with intermittent shortness of breath. Physical examination revealed diminished breath sounds in the lower third of the left chest. A chest X-ray showed a marked elevation of the left diaphragm (Supplementary Figure 1). An upper gastrointestinal tract X-ray revealed a mesenteric axis gastric volvulus (Supplementary Figure 2). A computed tomography (CT) scan showed the posterior part of the left diaphragm ballooning into the thoracic cavity, accompanied by the compression of the left lung (Supplementary Figure 3). Finally, the patient received the three-step procedure of laparoscopic diaphragm reconstruction. X-ray and CT on postoperative day 5 showed that the left diaphragm had returned to its normal anatomical position without signs of gastric volvulus (Supplementary Figure 4). Subsequently, the chest drain was removed, and no postoperative complications were observed. The patient was discharged on postoperative day 7 and remained in good health at 7-month follow-up.

## Surgical approach

We used the standard laparoscopic technique with five trocars. The patient was placed in a reversed trendelenburg position. The port placement is shown in Supplementary Figure 5.

### Step 1: to balance the pressure between the thoracic and abdominal cavities

**Figure 1.**
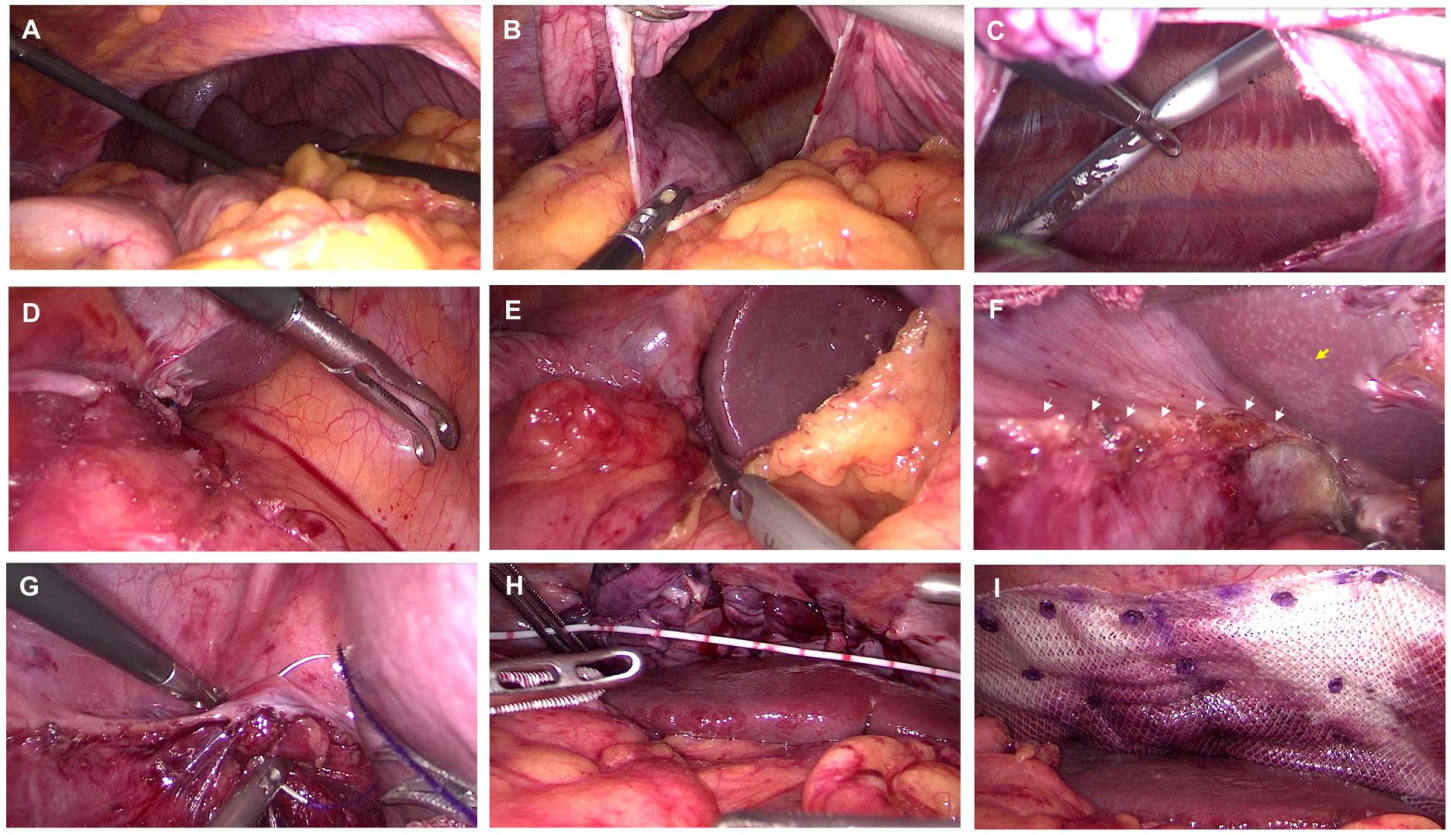
Surgical procedure of the diaphragm reconstruction. (A) The posterior part of the left diaphragm ballooning into the thoracic cavity, forming a sac-like space, with multi-organ herniated into it. (B) Incise the diaphragm to balance the pressure between the thoracic and abdominal cavities. (C) Place a drainage tube into the left thoracic cavity and connect it to a regular drainage bag. (D) and (E) Dissect the splenorenal ligament, splenophrenic ligament, and the splenogastric ligament. (F) Dissect the boundary of the defect. Yellow arrow, the spleen. White arrows, the inferior boundary. (G) Resect the weakened and redundant diaphragm, and reconstruct it using continuous suturing. (H) Measure the size of the defect. (I) Reinforce the diaphragm with a mesh.

As shown in Figure 1A, in cases of left posterior diaphragmatic eventration, the weakened diaphragm elevates abnormally into the thoracic cavity, forming a sac-like space. Organs like the stomach and spleen are often pulled into this space. First, we made a small incision in the elevated diaphragm and then extended this incision (Figure 1B). This can balance the pressure between the thoracic and abdominal cavities. In this way, the stomach and other organs can be easily pulled back. Since the lung tissue is often compressed in DE, most patients will undergo pleural effusion after operation. Thus, we made a small incision between the seventh and eighth ribs and placed a chest drainage tube (Figure 1C). Additionally, we observed that the expanded diaphragm was composed of peritoneum, muscle, and pleura (Figure 1C). This was consistent with the characteristics of DE described by Carter *et al*. [2].

### Step 2: to dissect the boundary of the defect

Before dissecting the boundary of the defect, it is crucial to dissect and reposition the spleen back into the abdominal cavity. The process is challenging due to the constraints imposed by the splenic ligaments. Therefore, we first divided the splenorenal ligament, the splenophrenic ligament, and the splenogastric ligament (Figure 1D and E). We then proceeded to dissect the inferior boundary of the defective diaphragm, creating enough space for mesh placement (Figure 1F).

### Step 3: to reconstruct the diaphragm

Since the diaphragm is weakened and redundant at the site of expansion, the excess diaphragm can be removed or sutured together through imbrication. In this case, we resected most of the redundant diaphragm and then reconstructed it using a slow-absorbing barbed suture with a continuous suturing method (Figure 1G). It is important to start the suture at the border of the diaphragm. This allows for a stronger fit. Finally, we measured the size of the defect (Figure 1H), placed an appropriate mesh for reinforcement, and secured the mesh with absorbable screws (Figure 1I).

## Discussion

Minimally invasive surgery is a widely used surgical approach in the treatment of DE. However, there are certain limitations in traditional methods. In cases of diaphragmatic plication and endostapler resection, it may not always be easy to fully eliminate the elevation of the weakened diaphragm. Additionally, during thoracoscopic diaphragmatic plication, the blind side of the abdominal cavity makes it impossible to avoid accidental injury of organs herniated into the thoracic cavity.

DE was initially considered as a specific type of thoracic hernia, although later it was defined as diaphragmatic eventration [6]. Thus, diaphragm reconstruction is likely to be the most ideal procedure for it. However, there are no prior records of using this method. In order to address the potential challenges of it, we first made an incision in the abnormal diaphragm to balance the pressure of the thoracic and abdominal cavities. This facilitates the retraction of the contents of the hernia and isolation of the spleen. When dissecting the lower boundary of the defect, the surgeon stands on the right side of the patient. This avoids the visual obstruction of the spleen and facilitates maneuvering. Overall, these measures make the process of diaphragm reconstruction easier to practice.

Gastric volvulus is the result of a disorder of the normal fixation mechanism of the stomach [7] and is often accompanied by DE [8]. Gastric volvulus can result in gastric perforation and often leads to a high mortality rate [9]. Preoperative imaging showed a mesenteric axis gastric volvulus. However, the patient had no gastrointestinal symptoms and no gastric volvulus was found during the operation. Interesting, in the procedure, we observed that the gastric fundus was pulled into the elevated cavity, with a noticeable upward shift in the position of the gastric antrum, which was very close to the esophagus. This may be the reason for the imaging findings that resemble gastric volvulus.

In conclusion, this study presents a new concept for the surgical treatment of DE from the perspective of hernia repair. This method seems to be more in line with the principles of mechanics. Of course, this needs to be further confirmed by future studies.

## Supplementary Material

goae043_Supplementary_Data
