# Supplementary material for: Laparoscopic management of diaphragmatic eventration: a three-step procedure of diaphragm reconstruction
Source: Gastroenterol Rep (Oxf). 2024 May 6;12:goae043. doi: 10.1093/gastro/goae043 (PMC11074006; doi:10.1093/gastro/goae043)
Supplement: goae043_Supplementary_Data [file goae043_supplementary_data.zip › Supplementary material.docx]

**Supplementary material**

**Supplementary figure 1.** Coronal (**A**) and sagittal (**B**) view of chest X-ray showed elevation of the left diaphragm.

**Supplementary figure 2.** Preoperative upper gastrointestinal tract X-ray showed a mesenteric axis volvulus of the stomach. (**A**) Coronal view. (**B**) Sagittal view.

**Supplementary figure 3** Preoperative computed tomography scan showed the posterior part of the left diaphragm ballooning into the left thoracic cavity, with abdominal contents herniated into it and volvulus of the stomach. (**A**) Coronal view. (**B**) Sagittal view. (**C**) Axial view.

**Supplementary figure 4.** Postoperative examination. Chest X-ray (**A & B**) and CT scan (**C & D**) showed the left diaphragm returned to normal, with no signs of gastric volvulus.

**Supplementary figure 5.** Distribution of the trocars.
