# Supplementary figures and images for: Laparoscopic management of diaphragmatic eventration: a three-step procedure of diaphragm reconstruction
Source: Gastroenterol Rep (Oxf). 2024 May 6;12:goae043. doi: 10.1093/gastro/goae043 (PMC11074006; doi:10.1093/gastro/goae043)

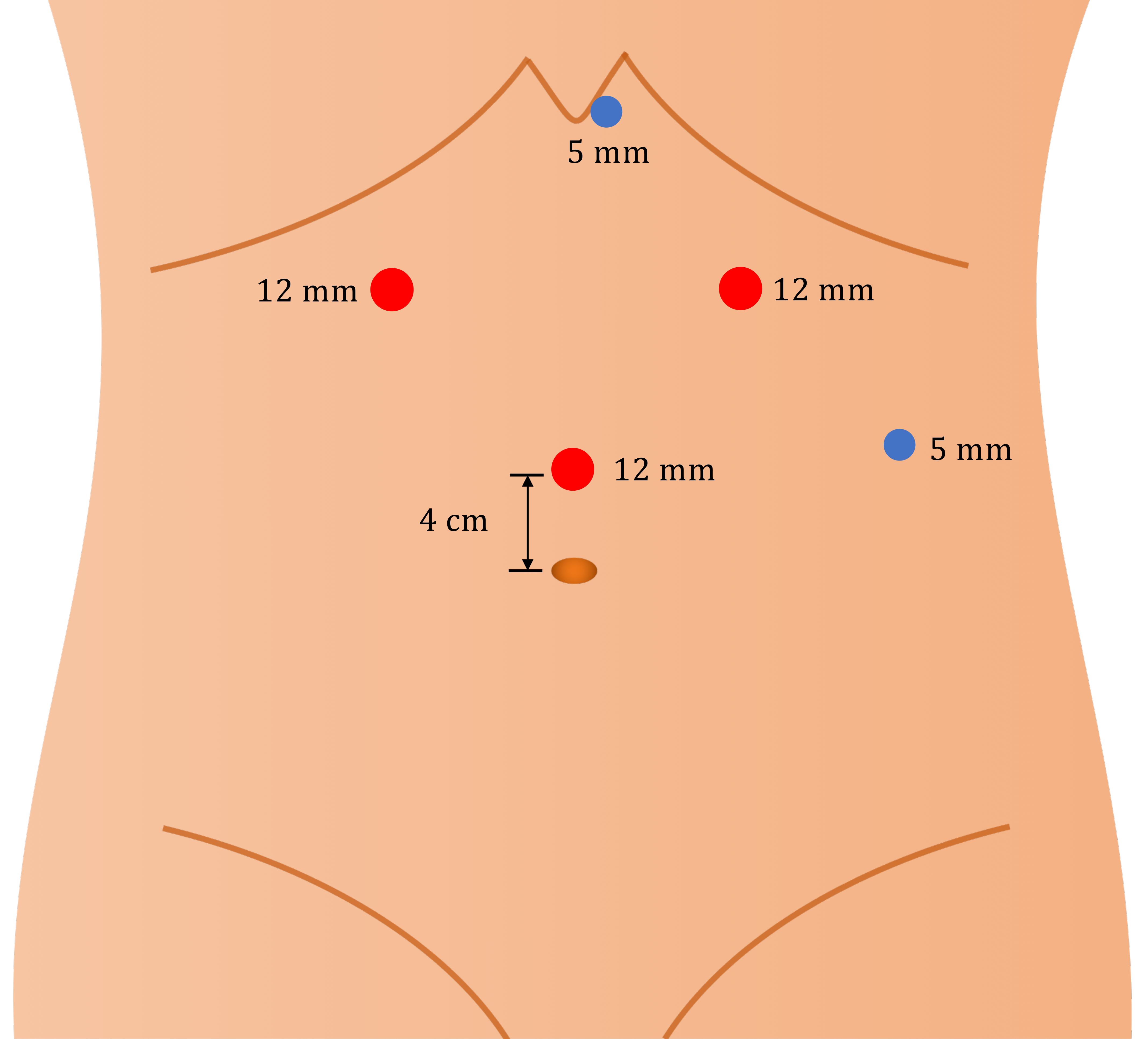

Supplement: goae043_Supplementary_Data [file goae043_supplementary_data.zip › Sup Figure 5.tif]

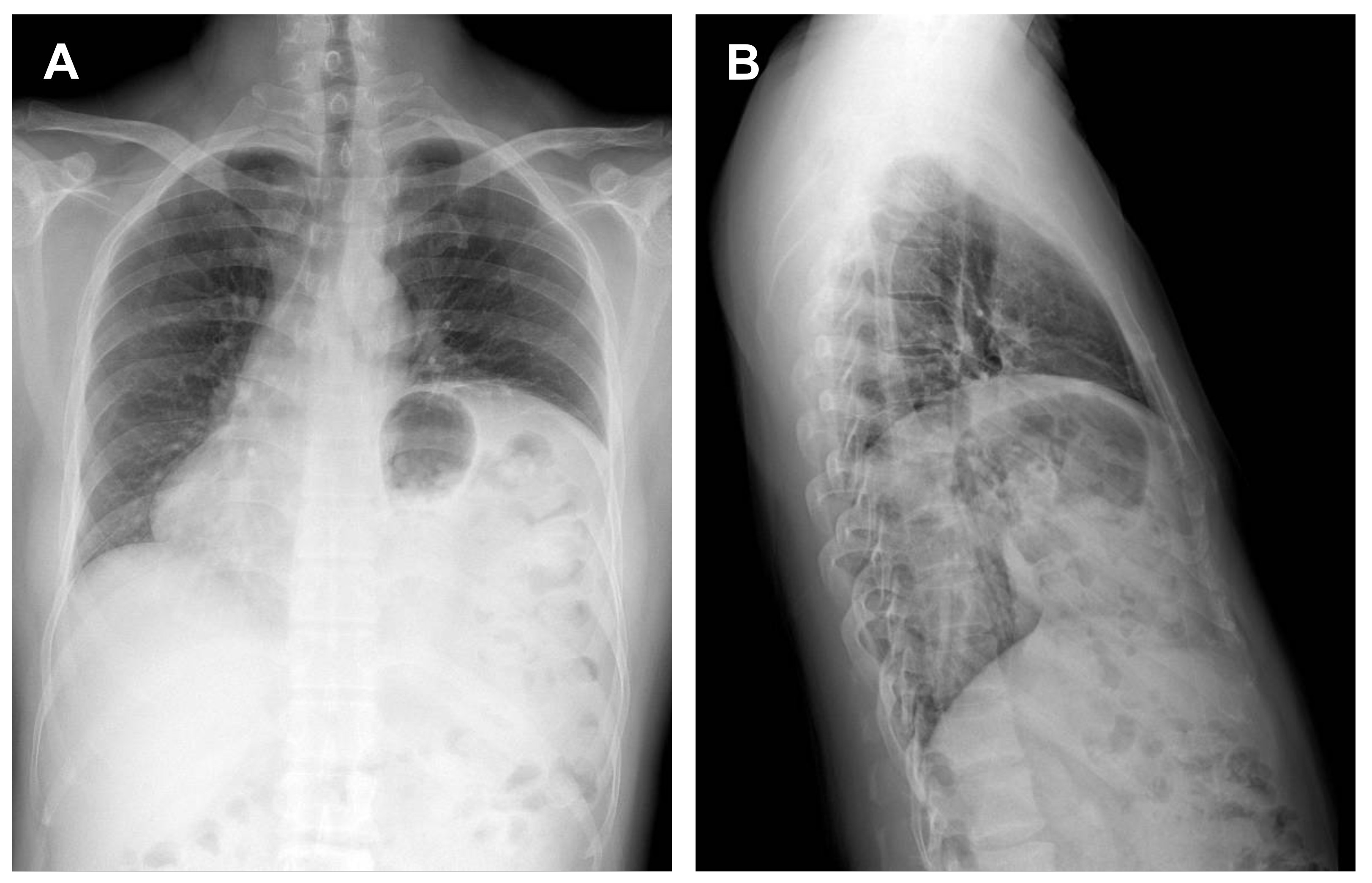

Supplement: goae043_Supplementary_Data [file goae043_supplementary_data.zip › Sup Figure 1.tif]

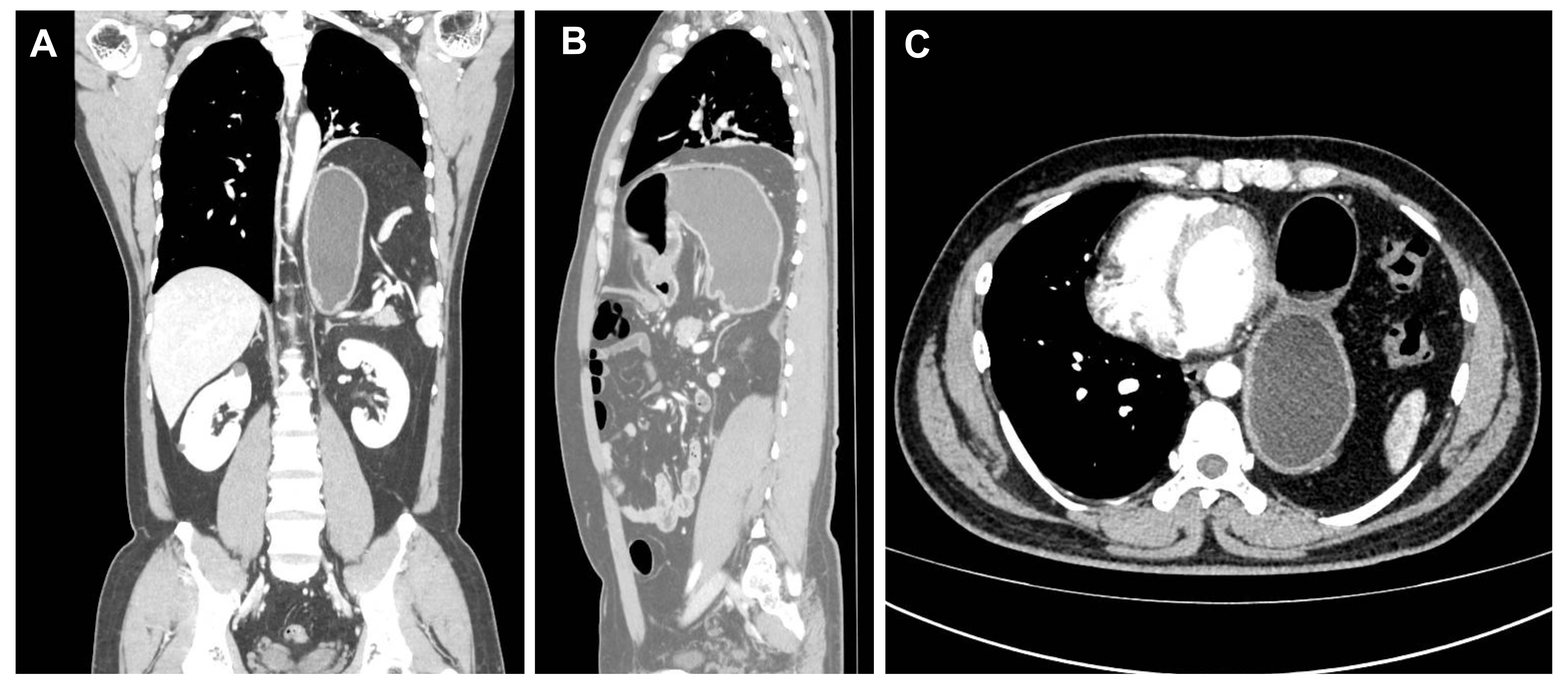

Supplement: goae043_Supplementary_Data [file goae043_supplementary_data.zip › Sup Figure 3.tif]

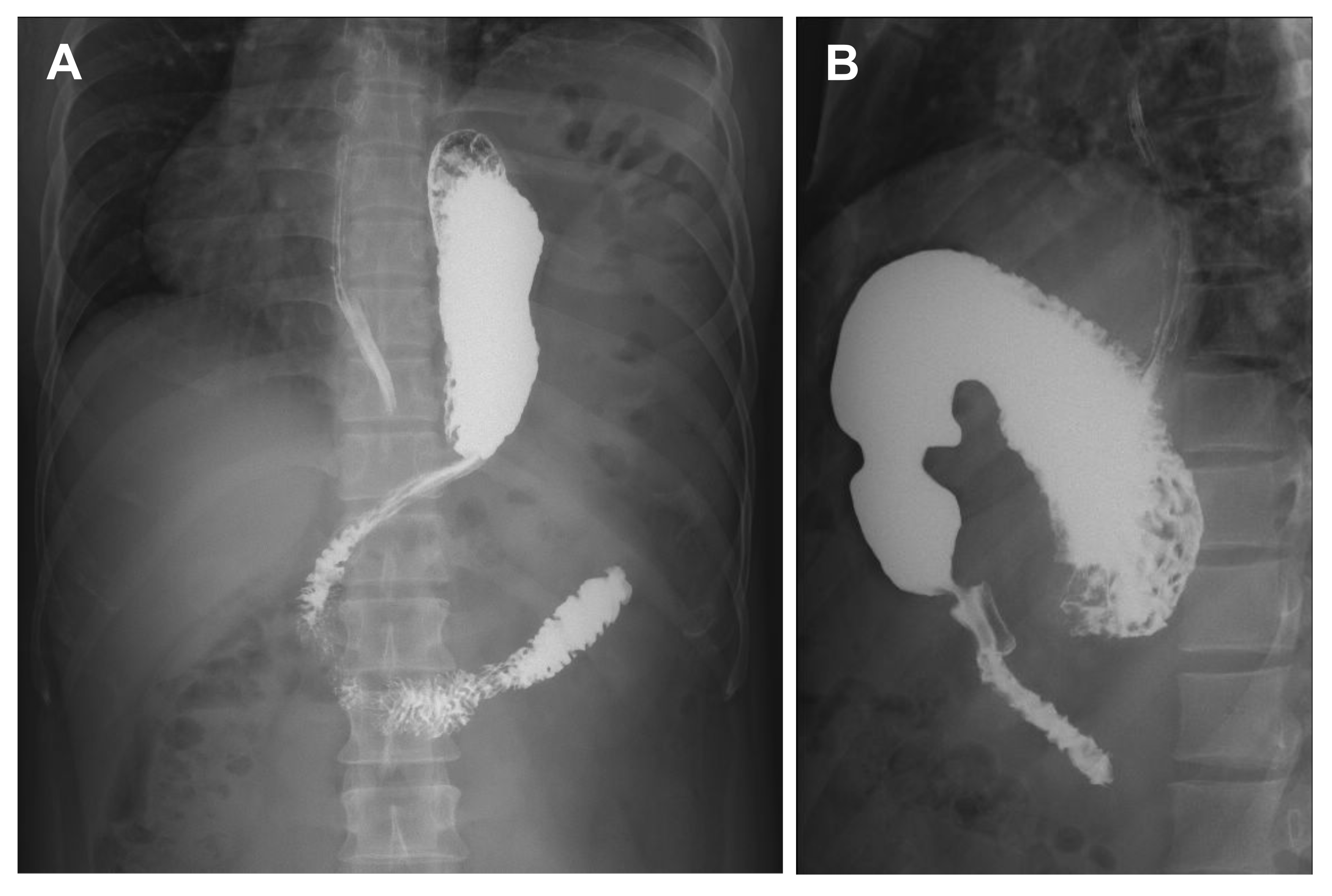

Supplement: goae043_Supplementary_Data [file goae043_supplementary_data.zip › Sup Figure 2.tif]

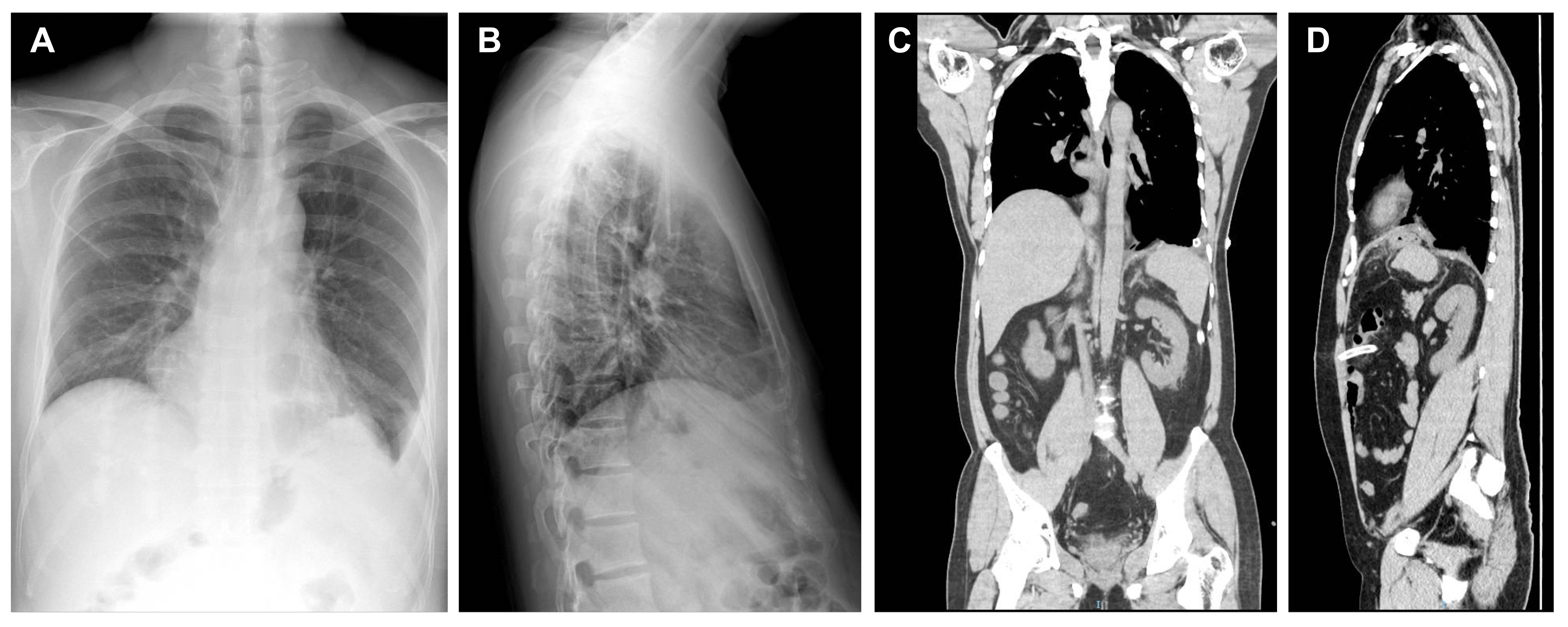

Supplement: goae043_Supplementary_Data [file goae043_supplementary_data.zip › Sup Figure 4.tif]
